# Supplementary material for: Effect of Ligilactobacillus salivarius and Other Natural Components against Anaerobic Periodontal Bacteria
Source: Molecules. 2020 Oct 2;25(19):4519. doi: 10.3390/molecules25194519 (PMC7582733; doi:10.3390/molecules25194519)
Supplement: Supplementary file 1 [file molecules-25-04519-s001.pdf]

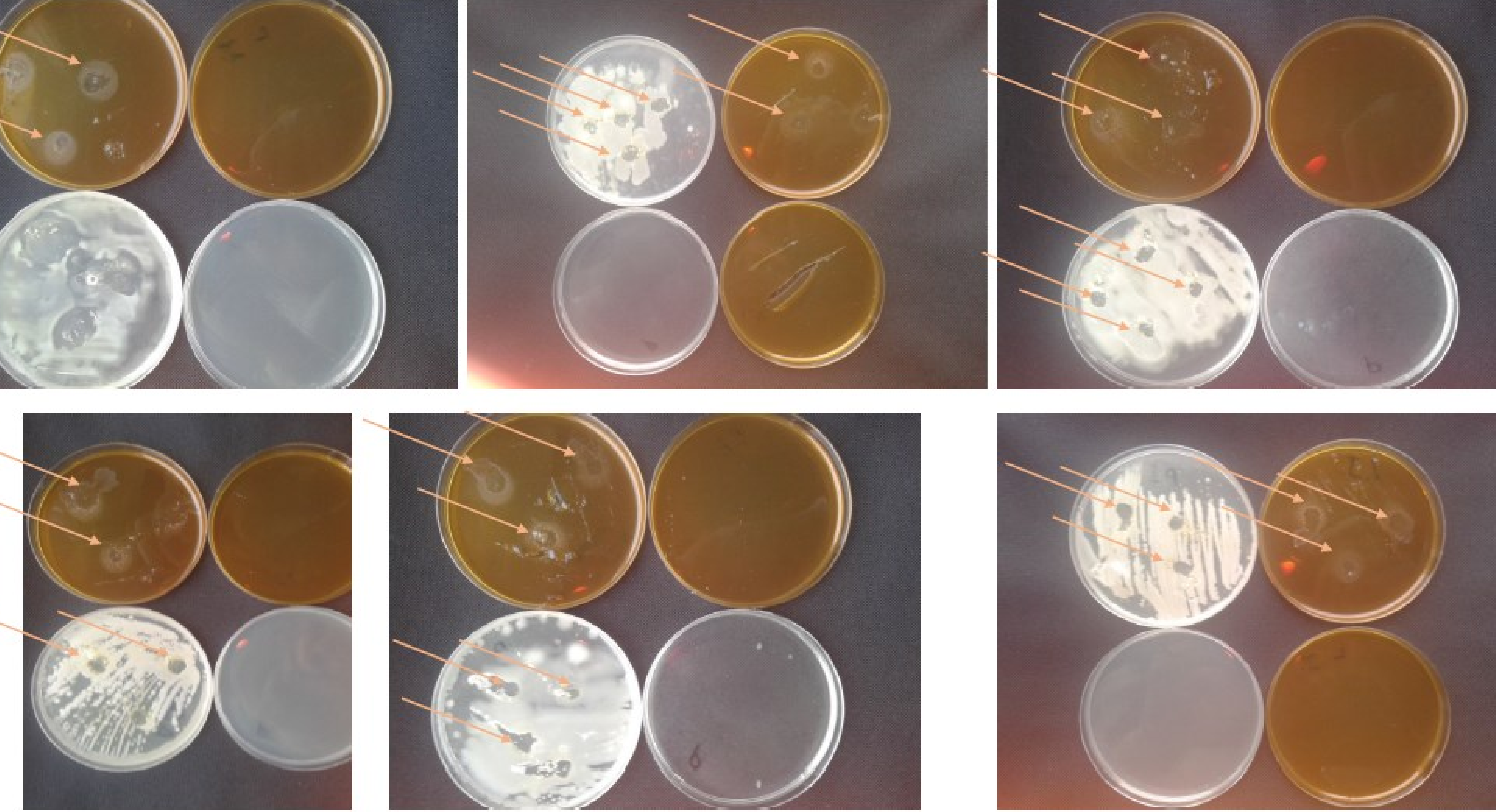

Figure S1. Zones of growth inhibition- Effect *in vitro* of Salistat SGL03 on oral microbiota. Yellow dish- universal medium, orange dish- selective medium
